# Medical and Philosophical Intelligence

**Published:** 1816-03

**Authors:** 


### MEDICAL AND PHILOSOPHICAL INTELLIGENCE.

*ROYAL SOCIETY.*—On Thursday, December 7, a paper by Dr. REID CLANNY was read, giving an account of his lamp for the security of colliers against fire-damp. He has constructed it of such a size that it may be put into the great-coat pocket. It may be made of copper for £1 14*s*. and of block-tin for 17*s*. A piece of mechanism at a low price is attached to the bellows, capable of supplying the lamp with air for an hour. Dr. Clanny relates a set of trials made in an apartment filled with carbureted hydrogen gas to the exploding point, and in a coal-mine the air of which was in the same state. In both cases the air within the lamp exploded, and the lamp was extinguished, but the external air was not in the least affected.

He shewed by a set of experiments, that by attending to the proper mode of supplying the lamp with air, the candle will continue to burn even when the carbureted hydrogen within the lamp explodes. Dr. Clanny states in this paper that the expence for steel mills in many collieries is much greater than would be requisite to light the mine by means of his lamp. In one mine, he says, it amounts to £30 a week. Dr. Clanny likewise gave an account

"* This idea first occurred to the acute mind of Mr. John Hunter."

of the numerous explosions that have taken place in the neighbourhood of Newcastle, and of the opposition which he has encountered in attempting to introduce his lamp into the coal-mines in the district in which he resides.

At the meeting, January 11, a paper by Sir H. DAVY was read, giving an account of a new method of preventing explosions of coal-mines from fire-damp. His method is to surround the flame of the lamp or candle with a wire sieve, the meshes of which amount to at least 250 in an inch. Such a sieve completely prevents the explosion from setting fire to the gas on the outside of it, even though the most inflammable mixtures of gases, as oxygen and hydrogen be present. This is certainly one of the most extraordinary and unaccountable facts connected with the propagation of heat and combustion. It is possible (supposing the fact to be correct) that so great an attraction may exist between the wires and the air surrounding them, that the internal combustion and expansion is not able to displace it. If we suppose such a fixedness to exist, it would account for the explosion not kindling the surrounding mixture on the outside of the sieve. This contrivance (supposing it effectual) would completely answer the purposes of the miner. Such sieves might be made for a halfpenny a-piece, and they would not in the least obstruct the light, or prevent the candle from being used by the miner as it is at present; whereas the bulk, and little light given out by the lamps, constitutes a serious objection to their use.

---

*Linnæan Society.*—On Tuesday, December 5, the remainder of Dr. ACHARIUS's paper describing two new genera of lichens was concluded.

A curious paper was likewise read, giving an account of the ancient inhabitants of Guadaloupe near the spot where the fossil human skeleton was found. Two different tribes existed, to whom the writer of the paper gives the names of Caribes and Galipees. About the year 1710 they quarrelled, and a battle was fought between them on the spot where the skeleton was found. The Galipees were routed, and disappeared in consequence, having no doubt emigrated. The author seems to conceive that the skeletons of the warriors slain in that battle were speedily encrusted with the calcareous sand of the place, and that this recently formed stone constitutes the rock in which the fossil skeleton was found.

On Tuesday, December 19, a paper was read endeavouring to explain the way in which the rock containing the Guadaloupe skeleton was agglutinated. It contained, likewise, an enumeration of the different species of shells and madrepores the fragments of which occur in the rock.

At the same meeting a paper by Dr. MACBRIDE, of South Carolina, was read, giving an account of the fly-catching qualities of the leaves of the *Saracenia flava* and *adunca*. These leaves constitute a kind of tube with an operculum at the top. They contain a saccharine charine liquid, which allures the insect. It lingers some time on the margin of the leaf, but at last ventures in, and is drowned in the liquid, being unable to make its way up the tube, which is beset with hairs pointing downwards, and preventing its escape. The number of flies destroyed by falling into these leaves is very great. They are sometimes placed in rooms for the purpose of getting rid of flies.

On Tuesday, January 16, 1816, a paper by M. Richard, of the French Institute, was read, containing a description of two new species of American plants, the *xylopia sericea* and *oxandra laurifolia*.

---

*Royal Institute of France.*—Greek, Latin, and French Edition of the Fifteen Books of the Elements, and the Book of the Data of Euclid, by M. Peyrard. Commissioners MM. Prony and Delambre, reporter.

The Class had already given its approbation to a complete translation of all the works of Euclid remaining to us. M. Peyrard, author of this book, had compared the twenty-three Greek manuscripts which are in the king's library. The result of this comparison was, that none of these manuscripts is entirely conformable to the Oxford edition; that this edition, which is considered the best, and which is without doubt the most beautiful, is only, as far as the Greek text goes, a copy of the edition of Bale, from which it has taken even the most obvious faults; that most part of the manuscripts offer variations which fill up some blanks or elucidate some passages of the two principal editions; but that in general all these manuscripts differ little from each other, but considerably from the oldest manuscript marked No. 190, and taken from the library of the Vatican, from which it was sent into France by M. Monge. The text in it appears more pure, more clear, less prolix, and more intelligible. M. Peyrard has attached himself chiefly to this manuscript, and has mostly followed it in the edition of the Greek text, which he has joined to his Latin and French translations.

At the request of his Excellency the Minister of the Interior, the Class has named a commission to examine the fidelity of the translation, and the merit of the numerous variations which M. Peyrard has introduced into the text, or rejected at the end of the work. Another commission of the class of history and ancient literature had been at the same time invited by the minister to consider the new translation relative to style and execution. The reporters of both classes, after several conferences, were of the same opinion respecting the utility of the new edition. The Class of sciences heard and approved a long report in which the edition of M. Peyrard was examined in the greatest detail, and the conclusion of which is, that this edition is evidently superior to every other, and that the author has done every thing in his power to render it worthy of appearing under the auspices of the king, to whom it is dedicated.

The

*Report of the City of London Truss Society.*

| | | |
|---|---|---|
| Patients relieved by this Society in | 1808 .......... | 227 |
| | 1809 .......... | 570 |
| | 1810 .......... | 813 |
| | 1811 .......... | 1094 |
| | 1812 .......... | 1666 |
| | 1813 .......... | 1800 |
| | 1814 .......... | 2064 |
| | 1815 .......... | 2473 |
| | | 10,707 |

Of these, 6 females each had umbilical and double femoral hernia, 1 female had large ventral and double femoral hernia, 5 females had umbilical and ventral hernia, 1 female had ventral and prolapsus uteri, 6 females had umbilical and single femoral hernia, 3 females had right inguinal congenital hernia, 2 females had left inguinal and right femoral hernia, 1 female had left femoral and right obturator hernia, 4 females had ventral and single femoral hernia, 1 female had double femoral and right inguinal hernia, 1 female had double inguinal and umbilical hernia, 1 female had right inguinal and ventral hernia, 1 female had left inguinal and left femoral hernia.

Eighteen males each had left femoral and right inguinal hernia, 3 males had left inguinal and left femoral hernia, 12 males had left inguinal and right femoral hernia, 7 males had double inguinal and right femoral hernia, 6 males had double inguinal and left femoral hernia, 4 males had right inguinal and right femoral, 2 males each had double inguinal and double femoral hernia, 2 males had double femoral and right inguinal hernia, 3 males had ventral and right inguinal hernia, 1 male had ventral and right femoral hernia, 2 males had ventral and left inguinal hernia, 1 male had ventral and double inguinal hernia, 1 male had left inguinal, umbilical, and ventral hernia; 1 male had umbilical and left inguinal hernia, 1 male had umbilical and left femoral hernia, 1 male had umbilical and right femoral hernia, 8 males each had umbilical and double inguinal hernia, 2 males had double femoral and right inguinal hernia; 528 patients had congenital hernia; 5 males had very large varicose veins on the abdomen.

---

*The Medical Benevolent Society.*—The distinguishing feature of this Society is, the advantage, during life, of the Medical Practitioner, who shall (from misfortunes, to which all are exposed in the most successful practice, and even in the vigour of health) become an object for benevolence.

The following are the fundamental principles upon which the Society is proposed to be founded:

Any physician, surgeon, or apothecary, residing and regularly practising his profession within England or Wales, is eligible to be admitted into the Society.

The Society to be under the management of a President, and of a Court of eighteen Directors, to be elected by the members at large, large, three of whom are to go out of office annually, in rotation, and the vacancies to be filled at a general court of members; and of three Treasurers.

Every member of the Society, being of the medical profession, shall be eligible to the direction.

After the formation of the Society, every candidate must be proposed by two members to the court of directors, and his admission be determined by ballot.

Every member, upon his admission, shall pay the sum of one guinea, besides his subscription.

A Benevolent Fund is to be formed, and supported by voluntary benefactions, and by subscriptions of any sum not less than a guinea annually. This fund may also be augmented from the bequests of the affluent.

Any member who shall have contributed to the Benevolent Fund, or to the Annuitant Fund hereafter-mentioned, to the amount of *twenty-five guineas* at one payment, or *thirty guineas* by subscriptions, may, if in distressed circumstances, although he may not have attained the age of sixty years, be relieved from that fund, at the discretion of the court of directors; but in no case shall such relief exceed the sum of 30*l.* in one year.

No disbursement shall be made from the fund of the Society, except for the necessary expences, until the Society has been established *ten* years.

All bequests, benefactions, and voluntary subscriptions, to be appropriated solely to the Benevolent Fund.

Contributors, who are *not* of the medical profession, to be considered as honorary members only; and to have no vote in the appropriation of the fund.

The accounts of the Society to be submitted to the members at certain general courts.

The laws for the future regulation of the concerns of the Society will be arranged when a court of directors is appointed, by whom, when digested, they will be submitted to a general court of subscribers, for approval and confirmation.

It having appeared, also, from the various communications which the committee has had the opportunities of receiving, that something in the way of an Annuitant Society would be extremely beneficial, Mr. Morgan, of the Equitable Assurance Society, was requested to draw up a table, which is annexed, in order to shew any medical gentleman at what rate of payment he may secure to himself an annuity, sufficient to place him above the pressure of want, when he is sixty years of age.

It is intended that every member who subscribes to the Annuitant Fund, according to the proposed scale of insurance, and upon otherwise conforming to the rules of the Society, shall have, at sixty years of age, an annuity, which will at least be 50*l.*; and that a certain part of the accumulated profits of the Annuitant Fund shall be applied, from time to time, at the discretion of the court of directors, to the increase of the annuities.

 It

It will be seen, by the table, that at whatever age a subscriber to the Annuitant Fund enters, he will have to pay annually, till he is sixty, the sum placed against his age in the column of *annual payments*; or, if he be disposed to subscribe, *at once*, the sum against his age in the column of *single payments*, he has nothing ever after to pay; and in either way, at sixty years of age, will be entitled, not by *benevolence*, but by *right*, to the annuity for the remainder of his life, provided he has been a subscriber ten years.

Many societies, for the same premiums, may offer as high an annuity; but the system of other societies is never to give a larger annuity than the one subscribed for. The plan here proposed is calculated to secure, not 50*l.* per annum only, but leads to the expectation of an annuity of much greater magnitude; because the profits arising from the payments will, as often as it can be safely done, be applied to the increase of the annuity:—thus, instead of 50*l.*, it may be double, or even more. Of course the Annuitant Fund, and the profits of it, must be strictly applied to the advantages only of the subscribers to it; and, as the directors of the Society will receive the subscriptions, and invest them in proper securities, hence little additional expence will be incurred in the management.

*Table shewing the Sum to be paid at one Payment, or the Sum to be paid Annually, in order to secure an Annuity of 50l. to the Subscriber during the remainder of his Life, after having attained the Age of Sixty.*

| AGE. | SINGLE PAYMENT. | | ANNUAL PAYMENT. | | AGE. | SINGLE PAYMENT. | | ANNUAL PAYMENT. | |
|---|---|---|---|---|---|---|---|---|---|
| | £. | s. | £. | s. | | £. | s. | £. | s. |
| 23 | 43 | 18 | 2 | 19 | 42 | 130 | 12 | 12 | 16 |
| 24 | 46 | 8 | 3 | 3 | 43 | 138 | 18 | 14 | 3 |
| 25 | 49 | 0 | 3 | 8 | 44 | 147 | 18 | 15 | 11 |
| 26 | 52 | 0 | 3 | 12 | 45 | 157 | 8 | 17 | 8 |
| 27 | 55 | 10 | 3 | 18 | 46 | 167 | 16 | 19 | 5 |
| 28 | 58 | 0 | 4 | 3 | 47 | 178 | 18 | 21 | 15 |
| 29 | 61 | 4 | 4 | 9 | 48 | 190 | 18 | 24 | 3 |
| 30 | 65 | 0 | 4 | 16 | 49 | 203 | 16 | 27 | 17 |
| 31 | 68 | 16 | 5 | 5 | 50 | 217 | 16 | 31 | 11 |
| 32 | 74 | 14 | 5 | 15 | 51 | 209 | 7 | 26 | 12 |
| 33 | 76 | 16 | 6 | 1 | 52 | 200 | 17 | 25 | 14 |
| 34 | 81 | 6 | 6 | 9 | 53 | 192 | 4 | 24 | 14 |
| 35 | 88 | 10 | 6 | 19 | 54 | 183 | 11 | 23 | 14 |
| 36 | 91 | 6 | 7 | 10 | 55 | 174 | 14 | 22 | 14 |
| 37 | 96 | 16 | 8 | 4 | 56 | 165 | 18 | 21 | 14 |
| 38 | 102 | 14 | 8 | 18 | 57 | 156 | 19 | 20 | 12 |
| 39 | 109 | 0 | 9 | 15 | 58 | 148 | 0 | 19 | 12 |
| 40 | 115 | 10 | 10 | 11 | 59 | 138 | 19 | 18 | 10 |
| 41 | 122 | 16 | 11 | 13 | 60 | 129 | 18 | 17 | 10 |

(Signed) WILLIAM MORGAN.

Any

Any person being above *fifty* years of age may subscribe to the Annuitant Fund, as well as to the Benevolent Fund.

The payments to be made from fifty to sixty years of age are now added.

---

Wednesday, the 14th of February, 1816.—This day being the anniversary of the late John Hunter's birth, was celebrated at the Royal College of Surgeons in the usual form. Mr. Cline, the master, was, according to custom, the orator on the occasion. His oration was throughout extempore; he began by remarking the great antiquity of the Art of Surgery, from the mention of it by Homer. From that time to the date of Hippocrates no writings appear to shew us the progress of surgery. We cannot, therefore, ascertain how far that venerable writer invented what appears under his name, or whether he collected the improvements of his predecessors.—A number of names are quoted by subsequent writers, but nothing worthy of notice has survived till Galen appeared. This Mr. Cline conceived might arise from the writings of Galen, containing the complete state of the art to his day, and thus lessening the importance of every thing that appeared between Hippocrates and himself. A just tribute was paid to Celsus, who it was observed collected most of his remarks from the Greek writers.

After this general view of ancient surgery, Mr. Cline described the great advantages derived by the moderns from a knowledge of human anatomy and the erection of public hospitals, all of which had become schools of surgery. An historical account followed of the two royal hospitals, St. Bartholomew's and St. Thomas's, and also of Guy's, the work of an individual during his life-time. The hospitals supported by subscription in London, and the principal county towns were also considered as sources of improvement in surgery. The orator next adverted to those characters whose writings were well known to the audience. Wiseman was the first in order, after whom he enumerated all those who had rendered themselves conspicuous in his (Mr. Cline's) hospital, beginning with Cheselden, and ending with Else his own immediate predecessor in the anatomical chair. He apologized for selecting only the name of Percival Pott from the other royal hospital, those of the Borough being his own acquaintance.

After paying every tribute of respect to that able surgeon and eloquent writer, he made a solemn pause before he introduced that illustrious name whose memory it was his duty on this occasion in a particular manner to respect. Premising a short history of his professional life, he first adverted to the commencement of his education at the age of twenty, under his brother Dr. William Hunter; this afforded an opportunity of descanting on the merits of that eloquent lecturer and accurate demonstrator. In pursuing the progress of J. Hunter, he described his services in the army, which never interfered with his physiological pursuits; he dwelt much upon the genius and industry of the man who, besides his important discoveries, had made seventeen thousand preparations in human and comparative anatomy, and to illustrate diseases in medicine and surgery. He remarked at the same time, that constant as his employment was, he had been often heard to say, that his greatest pleasure was *to think.* After many other equally judicious and pointed encomiums, he again felicitated his country on the number of its public hospitals, and, most of all, on the freedom of its constitution, which enabled every one to present to the public his opinions, his discoveries, and his improvements. Without this fortunate concurrence of events, Mr. Hunter might never have acquired his knowledge, or might have been prevented from its communication.

The oration was given throughout in flowing and rounded periods, in eloquent and perspicuous language, and with great animation. The theatre was of course crowded, but the greatest order prevailed, —Sir William Blizard particularly enjoining that no sounds of approbation should be expressed.

---

The following interesting little case (on which we venture a few remarks) is extracted from the last Number of the Repository.

"A. B. a healthy young man, complained of a swelling and pain in the right side of the tongue. I examined it, but felt by no means certain what was the nature of the swelling. In a few days, there was an evident fluctuation; and, upon plunging the lancet into the tumour, a discharge of well-formed pus flowed. I observed that the tumefaction did not extend beyond the central line of the tongue; the other side being of the natural size and appearance. The abscess healed, and my patient was discharged well.

"However, in a few days after, he called again, and complained of a similar swelling and pain on the left side of the tongue. On examination, it presented the same appearances as the other side on his first application. It preserved precisely the same course, was confined to that side only, suppurated, was opened, discharged about the same quantity of pus, and as soon healed."

Though we are not perfectly satisfied of the certainly of the conclusions drawn from the above case, yet we are willing to admit it among the collateral proofs that speech, as well as hearing and seeing, is endowed with double organs, no otherwise dependant on each other than from habitual sympathy. Our only objection is, that two distinct abscesses might be formed on the surface of, or even in the substance of the same muscle, divided only by the adhesive inflammation of Mr. Hunter; but this we mention merely to shew that further proofs are wanting, not to lessen the force of the above as far as it goes; and, as in the present instance, an important practical inference may be drawn, we are willing to give the argument all the force it will admit, and to strengthen it by the cases which introduce our inferences.

After many cases of hemiplegia, it is found that every power is recovered, excepting the speech. Perhaps, when we speak of a perfect recovery, our language may be, on most occasions, too strong.

 The

The paralytic limbs may recover sufficiently for the common purposes of life, and, if the eye or ear has been affected, its imperfect recovery may gradually be supplied by the improved power of the corresponding organs, or by their proving sufficient for all the wants of the patient. But the sympathies between the two sides of the tongue must be so early confirmed, so perpetual, so instantaneous, and so invariable, that the slightest incapacity on one side must very much embarass the free use of the whole organ, because one side cannot remain torpid whilst the other is acting. Hence we find men whose intellect, and, as far as we can judge, whose senses and muscular powers seem quite recovered, rarely recover their original powers of speech, if they have been at all paralysed. The elocution is not only slower and more embarassed, but they actually seem to speak as if they had something superfluous in their mouths, and something which impedes the free motion of the tongue.—Would it, therefore, if, after a careful examination, less sensibility should be found on one side of the tongue than on the other, be desirable at least to explain to the patient an opinion of the cause of his remaining inability to express himself, and leave it to his decision, whether so much of the paralysed side shall be taken away as may enable him to move the remainder with as little impediment as possible?—*Edit. of the Lond. Med. Journ.*

---

*An American Indian Warm Bath, which was found successful in a Case of Rheumatism that resisted the usual Remedies.*—For this purpose, a hole, about four feet deep and three in diameter, was dug in the earth, and heated well by a large fire in the bottom of it. The fire was then taken out, and an arch formed over the hole, by means of willow poles, and covered with several blankets, so as to make a perfect awning. The patient, being stripped naked, was seated under this on a bench, with a piece of board for his feet; and, with a jug of water, we sprinkled the bottom and sides of the hole, so as to keep up as hot a steam as he could bear. After remaining twenty minutes in this situation, he was taken out, immediately plunged twice in cold water, and brought back to the hole, where he received the vapour bath. During all this time he drank copiously a strong infusion of horse-mint. At the end of three quarters of an hour, he was again withdrawn from the hole, carefully wrapped, and suffered to cool gradually. This operation was performed yesterday; and this morning he walked about, and is nearly free from pain.—*Lewis and Clarke's Travels to the Source of the Missouri River, &c.*

---

A M. Massa, a modeller in plaster, is exhibiting at Paris the heads of famous capital felons. This is one of the results of the lectures of the renowned Dr. Gall, who seems to have excited a much stronger sensation in France than in London.—*Times Newspaper.*

On the above we cannot help regretting the manner in which Spurzheim has been used in some of the periodical journals of this town. A man of superior intellect, who has discovered and demonstrated what was never before suspected in that important organ, the brain, and whose discovery has been adopted by Sir Everard Home, V. P. of the Royal Society, in a paper read before that learned body, has been treated, not with contempt,—we can hardly use so gentle a term as obloquy,—but with the coarsest abuse, for opinions, some of which are certainly but ill supported. But should not common candour, not to say decency, have given some credit to a professor, a man of science, a scholar, and a free communicator, for a most important discovery, the novelty of which may have betrayed him, like other discoverers, into some eccentricities.

---

The Hottentot Venus, it appears from the French papers, died in Paris last week, after an illness of eight days. Her malady is said to have been the small-pox, which the physicians mistook successively for a catarrh, a pleurisy, and a dropsy of the chest. The Professors of the Museum of Natural History have procured the body, and are dissecting it for the gratification of the curious, or, as they term it, for the benefit of science. *Bell's (Sunday) Weekly Messenger, Jan.* 7, 1816.

We have since heard this account confirmed by one who was the companion of her voyage from Africa. From the same source we learn, that she often expressed a great horror of the small-pox, which always proved dreadfully fatal whenever it invaded her district. On her first arrival in London, when she was seen by the Prince Regent, in company with Dr. Baillie, the latter strongly importuned her to be vaccinated, but without success.

---

The following is our medical intelligence from the Continent. Amongst it will be found some interesting articles. If others are less so, our readers may be assured that we have not been negligent in our selection.

Dr. Frank, in Vienna, relates the following case of a gardener's wife near Vienna, who, after having been tormented for seven whole years with an almost uninterrupted and very painful head-ach, at last had been relieved by a lucky chance. She was twenty-four years of age, not subject to any kind of sickness, when she began to be seized with a very troublesome and frequent returning head-ach, which gradually became more violent, drove her almost to despair, and extended over the whole head, even to the *maxilla inferior.* This head-ach was intermittent; sometimes the patient suffered uninterruptedly for two or three months, and at others the pain was but slight. During this period, the patient not only felt a dryness in the nostrils, but also a very troublesome sensation of an entire stoppage in those parts. The physicians being now of opinion that all possible remedies had been exhausted, she was advised by one of them only to take a pinch of snuff frequently. This soon caused a very moderate secretion of phlegm, for which reason the the patient resolved to heighten the irritative power of the snuff, by mixing it with a little marjoram and assafœtida, both which articles she had in the house. Soon after the use of this sternutatory, on blowing her nose, a living worm dropped out, which, according to her description, perfectly resembled the common grub. The complaint still continuing with equal violence, she concluded, perhaps, still more worms might exist, and therefore resolved to increase the portion of assafœtida in the snuff, when, soon after, five worms more, similar, in every respect, to the above, issued from the nose; and some days after, three more made their appearance; and, in short, forty-eight worms were gradually voided through the nose, then followed a vast quantity of phlegm, and even several pieces of pseudo-membranes: the head-ach was, for the most part, gone; only a painful sensation remained for some time, which, however, some months after, quite disappeared. Dr. Frank is inclined to suppose these worms had their seat in the sinus frontales, in the two antra highmoreana, and the cavum naricum, which supposition seems to be, in some measure confirmed by the patient's feeling much pain in the ossa frontis, which induced her to take the resolution of having several teeth successively drawn. He also thinks the constant irritation of the worms in these parts might have brought on a chronic inflammation, productive of the pseudo-membranes voided after the worms.

According to the most recent observations of Dr. MURSINNA, at Berlin, the efficacy of the application of bark, combined with cinnamon, in intermittent fevers, immediately before the fit is coming on, which was recommended by Dr. Nasse, has again been experienced. Mr. Mursinna treated a lady suffering, for a considerable time, under a larvated [obscure] intermittent fever, with a variety of remedies, even with the Solutio arsenicalis, though without any effect. Many relapses having taken place, Mr. M. prescribed half a drachm of bark, with ten grains of cinnamon, to be taken in wine and water, directly before the attack, which was now much shorter than before. Next day the same dose was repeated, just before the fit was expected, which was not only of very short duration, but became almost imperceptible. After the third dose, not the least attack of the fever succeeded.

Prof. KERN, at Vienna, endeavours to prove, that the operation of piercing the tympanum can only produce the wished for effect in such cases where deafness is occasioned by a too thick tympanum, immoveable ossa auditus, an entire want of the same; by a too thick immoveable foramen ovale, or rather of the membranes shutting the same; or by a stoppage of the tuba Eustachii. In every other case where deafness originates in a faulty formation of the organs that are to receive the impression caused by the vibrations, or are to reflect this impression when received, the piercing of the tympanum cannot be of any avail. However, as it is but rarely hurtful, this expedient may be resorted to in dubious cases.

[The remaining articles in our next.]

Mr.

Mr. Fox, Surgeon-Dentist, Argyle-street, will commence his Lectures on the Structure and Diseases of the Teeth, on Friday, March 1st, at half-past five o'clock in the evening, in the Medical Theatre, Guy's Hospital.

Mr. Clarke will commence his next Course of Lectures on Midwifery and the Diseases of Women and Children, on Monday, March 18th. The Lectures are read every morning from a quarter past ten to a quarter past eleven, for the convenience of students attending the Hospitals.

### REPORT OF DISEASES.

The town has scarcely recovered from the sudden frost, and as sudden thaw. Catarrhs have been nearly as obstinate as during the severest winters, and have an highly inflammatory aspect, even among the oldest and apparently most debilitated. Children suffer exceedingly with hooping cough, scarlatina, and measles. Small-pox has greatly subsided: probably the late alarm has induced mothers to adopt vaccination with less scruple, and, consequently, with less delay. Phrenitis among children, if not more common, is certainly more noticed. Some cases have fallen under the inspection of the Reporter, in which the antiphlogistic plan was pursued with due vigour in the commencement, yet they proved fatal: others have been relieved by the same treatment. If such children are old enough to be blooded by a vein, a prognosis may often be formed, by the appearance of the blood. If the symptoms are severe, and the blood coagulates imperfectly, leaving a large portion of loose crassamentum, we can have but little hopes. Still, however, we have no remedy but bleeding, free doses of calomel, and semicupium. Blistering, if used at all, should be at a distance from the head.

All the above remarks are applicable to croup, which is only high inflammation in a different part, and which has occurred very often during the two spring months of the present year. Ophthalmia has not been less violent, especially among children, and required the same bold treatment to preserve an organ so important for beauty and use. In one case of this kind, in a girl not more than twelve years old, it was found necessary to renew the application of four leeches three times in twenty-four hours, though the subsequent bleeding was considerable each time, and though cathartics were administered with the same freedom.

Dyspepsia, with apparently a chronic inflammation of the stomach, has been more frequent than usual. Some cases of two or three years standing have been relieved by repeated gentle bleedings. Alkalies, particularly magnesia, have been found very serviceable, with aromatics, and daily portions of warm water, taken like the Bath waters.

Typhus, of which we hear much in the neighbouring counties, is at present scarcely known in London.

Meteo-

### METEOROLOGICAL REGISTER.

### *From January the 25th, to February the 26th,* 1816.

Kept by C. BLUNT, Philosophical Instrument Maker, No. 38, Tavistock-Street, Covent-Garden.

| Moon. | Day. | Wind. | Barometrical Pressure. Max. | Min. | Mean. | Temperature. Max. | Min. | Mean. | |
|---|---|---|---|---|---|---|---|---|---|
| | 26 | NE | 29·42 | 29·35 | 29 38 | 50 | 34 | 42 25 | Rain |
| | 27 | N | 29 82 | 29 66 | 29 74 | 48 | 27 | 38·5 | Fair |
| | 28 | N | 29·90 | 29 89 | 29·50 | 47 | 21 | 37·25 | Fair |
| ● | 29 | N | 30·40 | 30·38 | 30·38 | 46 | 16 | 33· | Fair |
| | 30 | N | 30·49 | 30·46 | 30·47 | 44 | 18 | 29·75 | Fair |
| | 31 | NW | 30 37 | 30·22 | 30·23 | 42 | 21 | 29· | Fair |
| | 1 | NW | 30·02 | 29 88 | 29·93 | 41 | 20 | 28· | Fair |
| | 2 | NW | 29 73 | 29·59 | 29·65 | 42 | 27 | 33·5 | Fog & Rain |
| | 3 | NW | 29 62 | 29·58 | 29 59 | 45 | 31 | 38·7 | Fair |
| | 4 | N | 29 57 | 29·56 | 29 56 | 44 | 32 | 39·25 | Rain |
| | 5 | NE | 29·52 | 29·50 | 29 51 | 46 | 32 | 39·5 | Fair |
| ◐ | 6 | NE | 29 22 | 29·12 | 29 13 | 47 | 28 | 36· | Snow |
| | 7 | NE | 29·40 | 29·12 | 29·23 | 40 | 17 | 29·5 | Snow |
| | 8 | NE | 29·54 | 29·42 | 29·48 | 30 | 11 | 21·75 | Fair |
| | 9 | NE | 29 74 | 29·72 | 29·73 | 25 | 9 | 18· | Fair |
| | 10 | NE | 29·88 | 29 76 | 29·82 | 24 | 10 | 18· | Fair |
| | 11 | NW | 30·00 | 29 89 | 29·45 | 33 | 15 | 21 75 | Fair |
| | 12 | N | 30·38 | 30·20 | 30·34 | 30 | 16 | 36·25 | Fair |
| ○ | 13 | N | 30 43 | 30·42 | 30·42 | 43 | 27 | 34·75 | Fair |
| | 14 | W | 30 45 | 30 43 | 30·44 | 42 | 30 | 36· | Fair |
| | 15 | W | 30·43 | 30·28 | 30·35 | 46 | 33 | 39·5 | Fair |
| | 16 | N | 30 05 | 29·86 | 29·95 | 45 | 31 | 38· | Fair |
| | 17 | N | 30·07 | 29 94 | 30· | 40 | 27 | 33·5 | Fair |
| | 18 | W | 30·12 | 30·08 | 30·10 | 39 | 28 | 33 5 | Fair |
| | 19 | W | 30·10 | 30· | 30 05 | 48 | 38 | 43· | Fair |
| ◑ | 20 | W | 30 14 | 30·10 | 30·12 | 48 | 35 | 41·5 | Fair |
| | 21 | W | 30·20 | 30·16 | 30·18 | 50 | 37 | 43 5 | Fair |
| | 22 | SW | 30·25 | 30·20 | 30·23 | 50 | 36 | 43· | Fair |
| | 23 | W | 30·32 | 30·22 | 30·27 | 50 | 32 | 41· | Fair |
| | 24 | NW | 30·21 | 30·19 | 30·20 | 47 | 35 | 41· | Rain |
| | 25 | W | 30·00 | 29·90 | 29·95 | 50 | 33 | 41·5 | Rain |

RESULTS.

Mean barometrical pressure of the month 30·065
Maximum 30·49, wind at N
Minimum 29·12, ——— NE

Mean temperature of the month 34·8 deg.
Maximum 50, wind at W
Minimum 9, ——— NE

*Scale exhibiting the prevailing Winds during the Month.*

| N | NE | E | SE | S | SW | W | NW |
|---|---|---|---|---|---|---|---|
| 9 | 7 | 0 | 0 | 0 | 1 | 8 | 6 |

| | Mean barometrical pressure. | Mean temperature. |
|---|---|---|
| From the last quarter on the 21st Jan. to the new moon on the 29th | 29·55 | 40·78 |
| ——— new moon on the 29th Jan. to the first quarter on the 6th Feb. | 29·79 | 34·22 |
| ——— first quarter on the 6th, to the full moon on the 13th | 29·78 | 25·71 |
| ——— full moon on the 13th, to the last quarter on the 20th | 30·14 | 37·8 |

MONTHLY

